# Further delineation of Wiedemann‐Rautenstrauch syndrome linked with *POLR3A*

**DOI:** 10.1002/mgg3.2274

**Published:** 2024-02-13

**Authors:** Amjad Khan, Bushra Al Shamsi, Maryam Al Shehhi, Amna A. Kashgari, Aaisha Al Balushi, Fahad A. Al Dihan, Mohannad A. Alghamdi, Abothnain Manal, Ana C. González‐Álvarez, Stefan T. Arold, Wafaa Eyaid

**Affiliations:** ^1^ Faculty of Science, Department of Biological Sciences (Zoology) University of Lakki Marwat Lakki Marwat Pakistan; ^2^ Institute for Medical Genetics and Applied Genomics University of Tübingen Tübingen Germany; ^3^ Alexander von Humboldt Fellowship Foundation Berlin Germany; ^4^ National Genetics Center The Royal Hospital, Ministry of Health Muscat Sultanate of Oman; ^5^ Child Health Department The Royal Hospital, Ministry of Health Muscat Sultanate of Oman; ^6^ Genetics Division, Department of Pediatrics, King Abdullah International Medical Research Centre (KAIMRC) King Saud bin Abdulaziz University for Health Science, King Abdulaziz Medical City, Ministry of National Guard‐Health Affairs (MNGHA) Riyadh Saudi Arabia; ^7^ King Abdullah Specialized Children's Hospital (KASCH) Ministry of National Guard Health Affairs Riyadh Saudi Arabia; ^8^ College of Medicine King Saud Bin Abdulaziz University for Health Sciences Riyadh Saudi Arabia; ^9^ Bioscience Program, Bioengineering Program, Biological and Environmental Science and Engineering Division King Abdullah University of Science and Technology (KAUST) Thuwal Kingdom of Saudi Arabia; ^10^ Computational Biology Research Center King Abdullah University of Science and Technology Thuwal Kingdom of Saudi Arabia; ^11^ Centre de Biologie Structurale (CBS), INSERM, CNRS Université de Montpellier Montpellier France

**Keywords:** Biallelic missense variants, consanguineous family, *POLR3A*, WES, WRS

## Abstract

Wiedemann‐Rautenstrauch Syndrome (WRS; MIM 264090) is an extremely rare and highly heterogeneous syndrome that is inherited in a recessive fashion. The patients have hallmark features such as prenatal and postnatal growth retardation, short stature, a progeroid appearance, hypotonia, facial dysmorphology, hypomyelination leukodystrophy, and mental impairment. Biallelic disease‐causing variants in the *RNA polymerase III subunit A* (*POLR3A*) have been associated with WRS. Here, we report the first identified cases of WRS syndrome with novel phenotypes in three consanguineous families (two Omani and one Saudi) characterized by biallelic variants in *POLR3A*. Using whole‐exome sequencing, we identified one novel homozygous missense variant (NM_007055: c.2456C>T; p. Pro819Leu) in two Omani families and one novel homozygous variant (c.1895G>T; p Cys632Phe) in Saudi family that segregates with the disease in the *POLR3A* gene. In silico homology modeling of wild‐type and mutated proteins revealed a substantial change in the structure and stability of both proteins, demonstrating a possible effect on function. By identifying the homozygous variants in the exon 14 and 18 of the *POLR3A* gene, our findings will contribute to a better understanding of the phenotype‐genotype relationship and molecular etiology of WRS syndrome.

## Graphical abstract

The affected individuals were examined by a local consultant geneticist and medical doctor at the Royal Hospital, Oman and KAIMRC, Riyadh Saudi Arabia. Detailed clinical features including age, gender, family history, and consanguinity were recorded. After clinical investigation and consent form, blood samples were collected, then whole‐exome sequencing was performed; and data were analyzed by a bioinformatician. Finally, we found a causative variant in three families and submitted to the journal of Gene Medicine for publication.

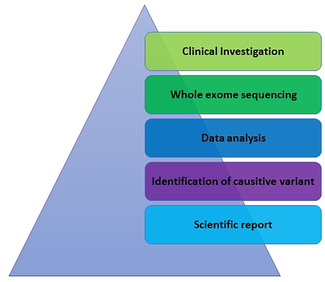

## 1 INTRODUCTION

Wiedemann‐Rautenstrauch Syndrome (WRS; MIM 264090) is classified as a group of rare genetic disorders mostly characterized by the intrauterine growth restriction (IUGR), triangular face, convex nasal or pinched nose, small mouth, widened fontanelles, low‐set ears, abnormal lower eyelids, pseudo hydrocephalus, prominent scalp veins, lipodystrophy, joint abnormalities, and hypodontia (Paolacci et al., 2017; Wambach et al., 2018; Wiedemann, 1979). The life expectancy in WRS is variable, as the condition might be life threatening and many affected individuals do not survive for long periods of time. The phenotypic spectrum of WRS and associated disorders is wide, and several genes have been associated with the condition [OMIM: https://www.omim.org/]. Genetic diagnoses are considered as one of the essential parts of medical genetics for prediction, prevention as well as prognosis or even treatment (Paolacci et al., 2017; Wambach et al., 2018; Wiedemann, 1979). Bi‐allelic variants in *POLR3A* have been associated with phenotypes distinct from WRS: hypogonadism, hypomyelinating leukodystrophy, and hypogonadotropic with or without oligodontia (Paolacci et al., 2017; Wambach et al., 2018; Wiedemann, 1979). Knowledge about the phenotypic spectrum associated with *POLR3A* variants and genotype‐phenotype associations are still limited and more cases from different ethnic populations may reveal novel insights to design treatment for such severe rare syndrome.

Here, we present the molecular analysis of Omani and Saudi Arabia consanguineous families by exome sequencing. We identified pathogenic variant in *POLR3A* genes (NM_007055: c.2456C>T; p. Pro819Leu and c.1895G>T; p Cys632Phe) segregating with the disease in an autosomal recessive manner.

## 2 MATERIALS AND METHODS

### 2.1 Study subjects

**FIGURE 1:**
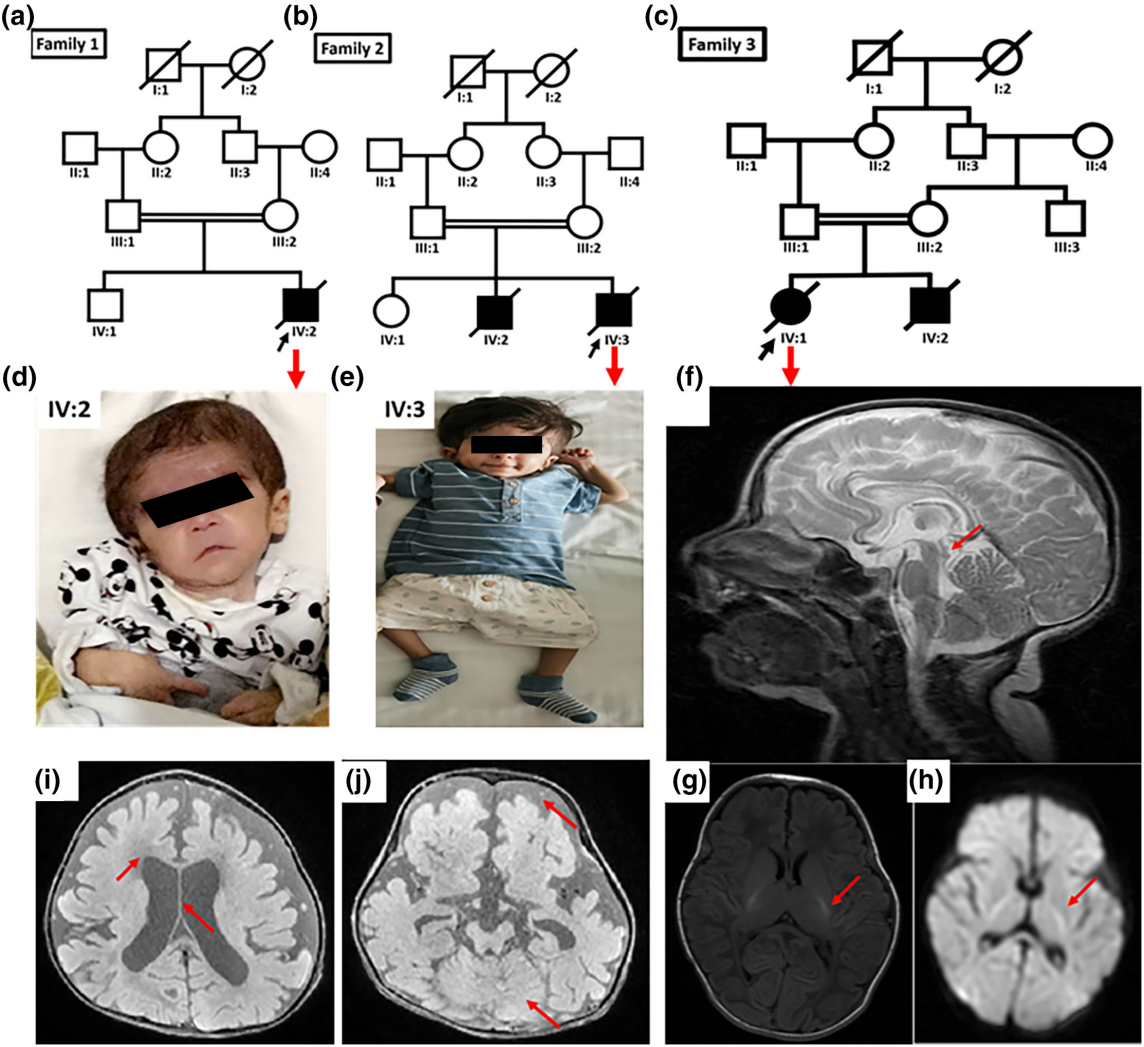
(a–c) Pedigrees of the present families. (d,e) Images of family 1 and 2 proband. (f–h) MRI of the proband (family 3), (f) Sagittal T2 WI images at 4 months old demonstrate progressive atrophy of the corpus callosum and the midbrain (red arrow) with abnormal T2 high signal intensity. (g,h) Axial T1WI and Axial T2WI Axial DWI and ADC (red arrow) abnormal TI high signal intensity in the posterior limb of the internal capsule with diffusion restriction in keeping with intramyelinic edema. (i,j) MRI of the proband (family 2). Brain MRI of (IV:3) at the age of 2 years showed diffuse cerebral atrophy with atrophied corpus callosum and posterior fossa (red arrows). There is delayed myelination.

The study design and protocol were reviewed and approved by the Ethical Review Committee (ERC) of the Royal Hospital, Sultanate of Oman, King Abdullah International Medical Research Center (KAIMRC, Riyadh, Saudi Arabia), as well as by the King Saud Bin AbdulAziz Health Sciences University. Written informed consent before data collections was obtained from all analyzed individuals. Pedigree was drawn (Figure 1a) and the affected individuals were examined by a local consultant geneticist and medical doctor at the Royal Hospital, Oman and KAIMRC, Riyadh Saudi Arabia. Detailed clinical features including age, gender, family history, and consanguinity were recorded.

### 2.2 Blood sample collection and DNA extraction

Genomic DNA was isolated from the peripheral blood obtained from affected members and available relatives using commercially available kits (QIAamp, Qiagen, Valencia, CA, USA). DNA quality was analyzed by agarose gel electrophoresis, and Nanodrop‐2000 spectrophotometer (Thermo Scientific, Schaumburg, IL, USA) was used for DNA quantification.

### 2.3 Library preparation and exome sequencing

Genomic DNA samples of affected individuals and their unaffected parents from each family were used to prepare the library for sequencing as described previously (Khan, Alaamery, et al., 2020). The generated libraries were submitted for whole‐exome sequencing at Centogene, the rare disease company (https://www.centogene.com). An Illumina platform (Illumina, San Diego, CA, USA) was used to obtain at least 20× coverage depth for >98% of the targeted bases.

### 2.4 Data interpretation

Centogene's end‐to‐end in‐house bioinformatics pipeline including base calling, alignment of reads to GRCh37/hg19 genome assembly, primary filtering out of low‐quality reads and probable artifacts, and subsequent annotation of variants was applied. Different variant interpretation and data analysis tools were used as previously described (Khan, Miao, et al., 2020; Shao et al., 2021). Briefly, we prioritized novel homozygous variants within the candidate autozygome of each index patient, and only considered coding/splicing variants that are predicted deleterious. All disease‐causing variants reported in the human gene mutation database (HGMD), ClinVar or CentoMD as well as all variants with minor allele frequency (MAF) of less than 0.1% in the gnomAD database were considered.

### 2.5 Computational structural analysis of mutants

To predict the three‐dimensional (3D) protein structure of *POLR3A* protein, the primary sequence was retrieved from the Protein Data Bank (PDB ID: 7AE3) models. Three‐dimensional models of wild‐type POLR3A^WT^ and mutant POLR3A^PRO819LEU^ and POLR3A^CYS632PHE^ proteins were manually inspected, and variants were evaluated using the PyMOL program (https://pymol.org/2/).

## 3 RESULTS

### 3.1 Clinical report

#### 3.1.1 Family 1

The members of family 1 live in the Dakhlia region in Oman. This four‐generation family includes one affected male (IV: 2) that were born from consanguineous parents at 37 weeks of gestational age. The pregnancy and delivery were unremarkable. Parents of the affected individual (IV: 2) are phenotypically normal and have no clinical symptoms related to WRS. The affected individual (IV: 2) was examined at the age of 2 months. His birth weight was 2.2 Kg. Clinical examination revealed anophthalmia, bilateral talipes equinovarus, seizures, global developmental delay, inguinal hernia, microphthalmia, nystagmus, recurrent infections, severe failure to thrive, Sinus tachycardia, talipes equinovarus, and vomiting. The patient died at the age of 7 months with severe complications. The family history on his father's side includes three cousins. Two of them had limb anomalies and died at an early age. The other male cousin is alive and has osteoporosis and learning difficulties. The laboratory workup including complete blood count (CBC), bone profile, and electrolytes test was all normal. The head and abdomen ultrasounds were also reported as normal after birth suggests that there are no major abnormalities in those areas as well. No cardiac, respiratory, digestive, and skin anomalies were observed. Additional clinical information of the affected individuals is summarized in Table 1.

**TABLE 1:** Clinical and molecular characteristics of families 1, 2, and 3.

Clinical findings	Family 1	Family 2		Family 3	
Gender	M	M	M	F	M
Age	2 months	6 months	2 years	7 months	2 years
Age of death	7 months	7 months	2 years	7 months	2 years
Ethnicity	Omani	Omani	Omani	Saudi	Saudi
Diagnosis	WRS	WRS	WRS	WRS	WRS
Mode of Inheritance	AR	AR	AR	AR	AR
Developmental delay	−	+	+	+	+
Cerebellar Signs	−	+	+	+	+
Microcephaly	−	−	−	+	+
Seizure	−	+	+	+	+
Low set ears	+	+	+	+	+
Pointed nose	+	+	+	−	−
Macroglossia	+	+	+	−	−
Epicanthal folds	−	+	+	−	−
Dysmorphic features	+	+	+	+	+
Skeletal problem	+	+	+	−	−
Optic atrophy	+	+	+	+	+
Brain atrophy	+	+	+	+	+
Nystagmus	+	+	+	+	+
Microstomia	+	+	+	+	+
Dermatitis anomalies	−	−	−	−	−
Hypodontia	−	−	−	−	−
Gene Name	*POLR3A*	*POLR3A*	*POLR3A*	*POLR3A*	*POLR3A*
Transcript ID	NM_007055	NM_007055	NM_007055	NM_007055	NM_007055
MIM number	614,258	614,258	614,258	614,258	614,258
Cytogenetic location	10q22.3	10q22.3	10q22.3	10q22.3	10q22.3
Nucleotide change	c.2456C>T	c.2456C>T	c.2456C>T	c.1895G>T	c.1895G>T
Protein change	p.Pro819Leu	p.Pro819Leu	p.Pro819Leu	p. Cys632Phe	p.Cys632Phe
GnomAD frequency	−	−	−	−	−
1000 G frequency	−	−	−	−	−
ESP frequency	−	−	−	−	−
SIFT	Deleterious	Deleterious	Deleterious	Deleterious	Deleterious
Polyphen2	Probably damaging	Probably damaging	Probably damaging	Probably damaging	Probably damaging
Mutation Taster	Disease causing	Disease causing	Disease causing	Disease causing	Disease causing
PROVEAN	Deleterious	Deleterious	Deleterious	Deleterious	Deleterious
ACMG Classification	PP3	PP3	PP3	PP3	PP3

Abbreviations: A, absence; AR, Autosomal recessive; F, female; M, male; WRS, Wiedemann‐Rautenstrauch Syndrome; +, mildly affected; ++, severely affected.

#### 3.1.2 Family 2

Family 2 also originates from the Al Sharqiya region of Oman and the parents are first cousins (Figure 1a). The family comprises two affected brothers (IV: 2 and IV: 3) out of three siblings, indicating an autosomal recessive inheritance. Clinical examination revealed global developmental delay, severe failure to thrive, seizures, global developmental delay, recurrent severe infection, arthrogryposis multiplex congenita, motor delay, spasticity, and triangular face with prominent forehead, microphthalmia, bilateral epicanthal folds, nystagmus, low set ears, microstomia, short neck and talipes equinovarus. The first affected child (IV: 2) had passed away at the age of 7 months due to aspiration pneumonia. The second child (IV: 3) developed a seizure at 2 years of age. His weight was 4.6 kg. An MRI of the brain report showed generalized cerebral atrophy with leukodystrophy. Brain MRI of (IV:3) at the age of 2 years showed diffuse cerebral atrophy with atrophied corpus callosum and posterior fossa. There is delayed myelination corresponding at less than 6 months (Figure 1i,j). Cystic area seen along the pyramidal tract in the brainstem with no diffusion restriction. Normal ventricles apart from ex vacuo dilatation secondary to the atrophy. An ultrasound scan showed short limbs (skeletal dysplasia) with hydrocele. Brain Magnetic resonance angiography (MRA) and magnetic resonance venography (MRV) were normal. Due to severe pneumonia infection the second child died at 2 years of age. Prenatal, perinatal, and neonatal medical records of both patients were normal. Additional clinical information of the affected individuals is summarized in Table 1.

#### 3.1.3 Family 3

The members of family 3 live in the Riyadh region of the kingdom of Saudi Arabia. This four‐generation consanguineous family includes two siblings, one female (IV: 1) and one male (IV: 2). The two children (IV: 1 and IV: 2) were diagnosed at the Department of Pediatrics, King Abdullah Specialized Children's Hospital (KASCH) Riyadh, Saudi Arabia. The pregnancy and delivery were remarkable. Both the children were born at 36 week of gestation and delivered via emergency cesarean. Parents of the affected individuals are phenotypically normal and have no clinical symptoms related to the disease. Detailed information including the parental marriage type, pedigree, disease history, affected and non‐affected subjects, and a number of sibships was obtained by interviewing family elders. On physical examination at one years of age, the proband measured 42 cm, weighed 1.90 kg and had head circumference of 30 cm. Clinical examination revealed with a history of seizures, speech delay, pneumonia, microcephaly, and dysmorphic features. The proband brother (IV: 2) is 6 months old, who presented the same symptoms of disease including severe seizure, mild microcephaly, and pneumonia. MRI investigation showed increased signal activity of the basal ganglia, progressive atrophy of the corpus callosum, and the midbrain, and an EEG showed multiple epileptiform discharges all over the brain (Figure 1f–h).

The family history on proband maternal side includes three cousins. Two of them had severe seizure and died at an early age. The proband brother (IV: 2) died at the age of 7 months with severe infection and seizures complication. Details clinical information is summarized in Table 1.

### 3.2 Molecular evaluation

**FIGURE 2:**
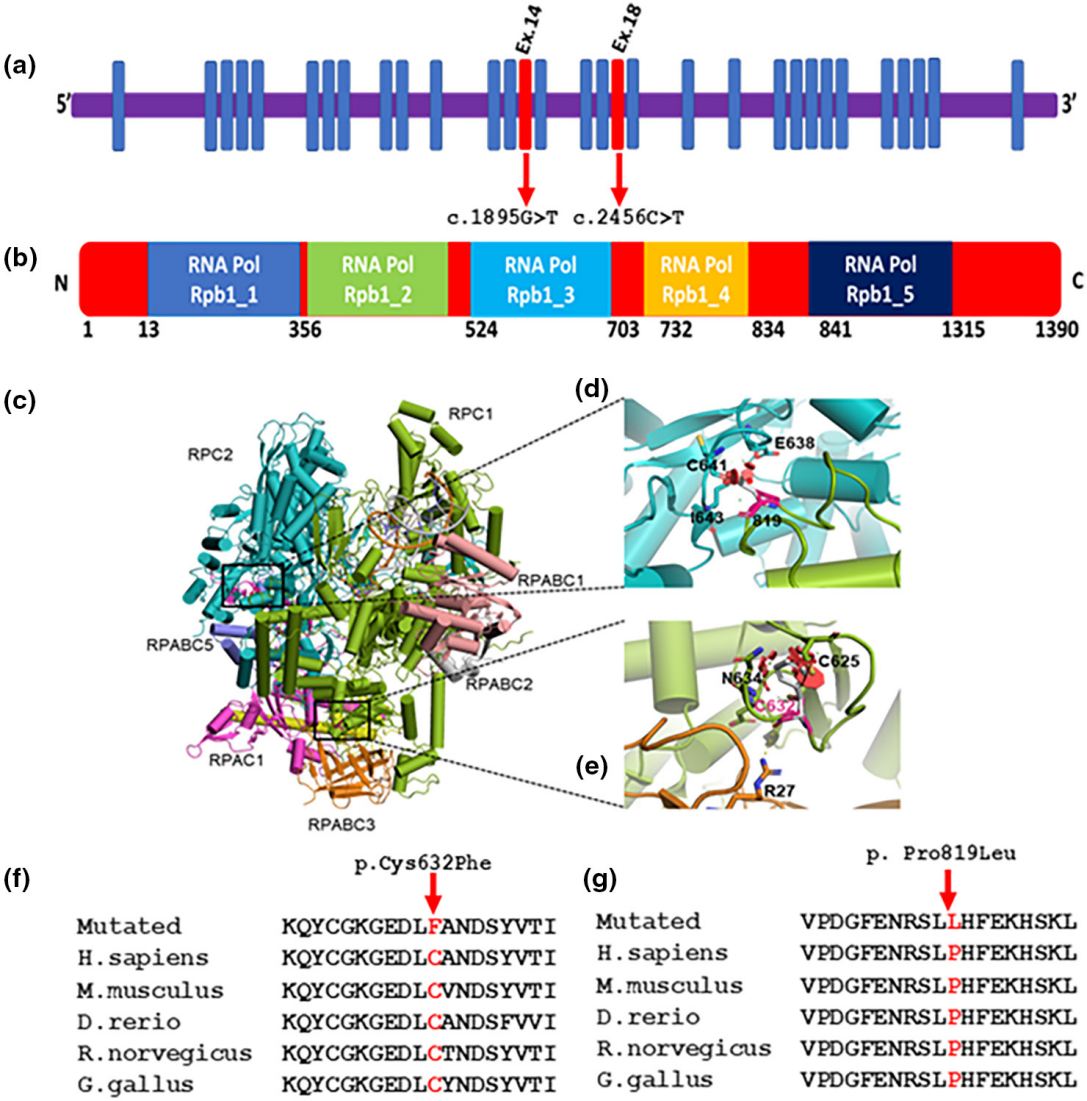
(a,b) Gene structure of 31 coding regions and protein domains of POLR3A. The exons in which the variants are identified are shown in red color. (c) Molecular environment and effect of the mutations in the RPC1 subunit of the RNA Polymerase III elongation complex 3. 3D representation of the core subunits of RNA Polymerase III illustrated by PyMOL with the crystal structure (PDB accession number 7AE3): RPC1 is shown in green, RPC2 in blue, RPAC1 in bright pink, RPABC1 in light pink, RPABC2 in gray, RPABC3 in orange, and RPABC5 in purple. (d) Close up location of the substitution of P819 shown in bright pink by Leucine shown in gray. The variant introduces clashes with amino acids I643, C641, and E638 from the RPC2 subunit. (e) Close up location of the substitution of C632 shown in bright pink by Phenylalanine shown in gray. The variant introduces clashes with amino acids C625 and N634 and is located at the interface with subunit RPABC3 shown in orange. (f,g) Conservation analysis of identified variants across different species.

By applying an iterative filtering strategy based on variant frequency, functional consequences on coding sequence and inheritance, one novel homozygous coding missense variant in the *POLR3A* gene NM_007055: c.2456C>T; p. Pro819Leu was identified in each index patient in both Omani patients and one novel homozygous variant was genes (NM_007055: c.1895G>T; p Cys632Phe) found in Saudi Arabia patient. The identified variants was not found in dbSNP (http://www.ncbi.nlm.nih.gov/SNP), Exome Variant Server (EVS, http://www.evs.gs.washington.edu/EVS), 1000 genome project (http://www.1000genomes.org), ExAC (http://exac.broadinstitute.org), or the gnomAD (https://gnomad.broadinstitute.org) databases. The *POLR3A* variants occurs at a highly conserved residue, and is predicted to be deleterious in all available bioinformatics results (Table S1). These variant genes (NM_007055: c.2456C>T; p. Pro819Leu and c.1895G>T; p Cys632Phe) occur in the exon 18 and 14 of *POLR3A* (Figure 2a). Both variants were highly conserved across various species (Figure 2d). The resulting protein if expressed would be predicted to be nonfunctional. These homozygous variants were confirmed to be present in affected children, while parents were heterozygous for the wild‐type allele.

### 3.3 Computational structural evaluation

With 1390 amino acids, POLR3A^WT^ is the largest of the 17 subunits that constitute the DNA polymerase III complex. POLR3A^WT^ forms the catalytic center of the enzyme along with POLR3B. POLR3A^WT^ has several domains (Figure 2b) that play different roles in positioning DNA for transcription. The clamp domain is located at the N‐terminus and is a mobile domain that positions DNA for transcription. Next is a domain that binds the magnesium ion at the active site of the enzyme, followed by the pore domain which serves as a channel through which nucleotides can enter the active site. The funnel domain contains the binding site for some elongation factors. Finally, the cleft domain is found at the end of the protein and is responsible for forming the central channel or cleft where DNA is bound (Girbig et al., 2021).

POLR3A^CYS632^ proteins are located in a loop region that connects two beta strands of the pore domain and connects this domain to the RPABC3 subunit. The backbone oxygen of POLR3A^CYS632^ proteins forms an H‐bond with the side chain of Arg27 of RPABC3 (shown in orange in Figure 2c). The substitution of POLR3A^CYS632^ proteins with the larger and non‐polar proteins Phe residue introduces clashes with nearby amino acids (Cys625, Leu630, and Asn634) introducing distortions in the loop region that would affect affecting the stability and conformation of the pore domain and its interaction with the RPABC3.

POLR3A^PRO819^ is in a loop that connects two alpha‐helices in the funnel domain. It is positioned at the interface with subunit RPC2 (marked in blue in Figure 2c) and directly contributes to the interface by forming an H‐bond with the side chain of Ile643 in the RPC2 subunit. The substitution of the rigid Pro residue by the nonpolar and larger Leu residue brings a hydrophobic moiety into close contact with backbone oxygens of residues Cys641 and Glu638. This could destabilize the loop and potentially affect the overall stability of the funnel subdomain. Both variants were predicted to undergo substantial structural rearrangements and impact on the stability of the complex by SIFT (Vaser et al., 2016) (Score = 1 untolerated substitution) and PolyPhen‐2 (Khan, Wang, et al., 2020) (Score = 1 probably damaging).

## 4 DISCUSSION

The *RNA polymerase III subunit A* (*POLR3A*) gene, also known as *ADDH; C160; HLD7; RPC1; WDRTS; RPC155; hRPC155*, is located on the q22.3 band of chromosome 10 and spans 54,367 bp. It is composed of 31 exons and has a coding sequence of 4170 nucleotides which encoded 1390 amino acids. It is constitutively expressed in all tissues but are highly expressed in the cerebellum part of the human brain (Bernard et al., 2011; Musumeci et al., 2022; Wolf et al., 2014). *POLR3A* coding for the largest subunit of RNA polymerase III, which is responsible for transcribing many small, non‐coding RNAs, such as tRNAs and 5S rRNA are critical for protein synthesis, as they are involved in the translation of mRNA into protein. Therefore, a down regulation in *POLR3A* expression could potentially lead to a decrease in the levels of these critical RNAs, resulting in dysregulation of protein synthesis (Musumeci et al., 2022; Wolf et al., 2014).

The mapping of pol III occupancy genome‐wide in the livers of different species, including mouse, rat, human, macaque, dog, and opossum, showed that the strength and location of pol III binding to individual tRNA genes can vary substantially between species (Kutter et al., 2011). *POLR3*‐related disorders caused by recessive mutations in the genes such as *POLR3A, POLR3B, POLR1C*, and *POLR3K* that encode subunits of RNA polymerase III [OMIM: https://www.omim.org/]. WRS is a heterogeneous disorder with many genes that have been implicated (Wambach et al., 2018; Wiedemann, 1979). Consanguineous families have a higher likelihood of carrying rare recessive genetic variants that can lead to disease. This is because consanguinity increases the probability of inheriting identical copies of genes from each parent, including deleterious variants online Mendelian inheritance in man [OMIM: https://www.omim.org/].

In this study, we report two Omani and one Saudi Arabia consanguineous families with WRS in an autosomal recessive pattern of inheritance, associated with homozygous *POLR3A* gene variants, (NM_007055: c.2456C>T; p. Pro819Leu and c.1895G>T; p Cys632Phe) previously described to cause WRS phenotypes (Moon et al., 2023; Paolacci et al., 2017; Wambach et al., 2018; Wiedemann, 1979). The affected patients presented with profound global developmental delay, generalized cerebral atrophy, bilateral talipes, triangular face, prominent forehead, small mouth, short neck, protruding tongue, small eyes with a pointed nose, microphthalmia, bilateral epicanthal folds, low set ears, flexion deformity at both thumbs with abnormal hand posture, seizure, and severe failure to thrive. Several families having homozygous or compound heterozygous mutations in the *POLR3A* gene have been reported with variable clinical manifestations (Atrouni et al., 2003; Saitsu et al., 2011; Timmons et al., 2006).

Paolacci et al. (2018) studied 15 patients from 12 families with WDRTS and identified compound heterozygous and splice site *POLR3A* variants in affected individuals (Paolacci et al., 2018). Another study in seven unrelated patients with WDRTS identified compound heterozygous mutations in the *POLR3A* gene that segregate in autosomal recessive manner (Wambach et al., 2018). Temel et al. (2020) studied a 5‐year‐old female which was born to non‐consanguineous parents with classical clinical features of WRS and found compound heterozygous for variants in the *POLR3A* gene (Temel et al., 2020).

Bernard et al. reported 19 patients having developmental delay, seizures, optic atrophy, nystagmus, dysphagia, hypersalivation, hypodontia, and hypogonadotropic hypogonadism with homozygous missense *POLR3A* mutations located in different domains (Bernard et al., 2011). Of 19 patients, one has the p. Met852Val mutation. This mutation is located in the cleft region of the polymerase on the bridge helix that connects the thumb and fingers domains of the RNA polymerase enzyme and help for stabilizing the polymerase during transcription and for positioning the template DNA strand and incoming nucleotides for correct base pairing (Bernard et al., 2011; Harting et al., 2020).

The variant identified in two Omani families also resides in the cleft region of the polymerase on the bridge helix domain is also associated with features including developmental delay, seizures, and brain and optic atrophy. However, some of the features such as bilateral talipes, triangular face, small mouth, short neck, protruding tongue, small eyes with a pointed nose, microphthalmia, bilateral epicanthal folds, low set ears, flexion deformity at both thumbs with abnormal hand posture, and failure to thrive was not observed in previously reported cases (Bernard et al., 2011; Harting et al., 2020; Musumeci et al., 2022; Wolf et al., 2014). In Saudi Arabia patients, there were seizures, speech delay, pneumonia, and microcephaly. Such phenotypes were not observed in previously reported cases in the human genome mutation database (HGMD: http://www.hgmd.org) and in OMIM (https://www.omim.org/).

To‐date around 172 disease‐causing variants in POLR3A gene have been associated to AR WRS with variable clinical features (HGMD: http://www.hgmd.org) (Supplementary table S1). It is worth noting that the clinical manifestations of *POLR3A* mutations can be highly variable, and some affected individuals may exhibit non‐neurologic features such as bone and limb abnormalities, while others may primarily have neurological symptoms (Bernard et al., 2011; Harting et al., 2020; Musumeci et al., 2022; Wolf et al., 2014; Yoon Han et al., 2022). The severity and specific symptoms of these disorders can vary widely, even among members of the same family. Clinical and MRI evaluation can be a useful tool for identifying characteristic features of the disorder, such as abnormalities in the cerebellum and brainstem.

Overall, the present study further supports previous findings that bi‐allelic variants in *POLR3A* cause WRS in AR manner. Our findings expand the knowledge on genotype‐phenotype correlations in WRS related to *POLR3A* variants. However, further research is needed to fully understand the pathogenesis of this disorder and to develop effective treatments for affected individuals.

## AUTHOR CONTRIBUTIONS

*Conceptualization*: Amjad Khan, Bushra Al Shamsi, Maryam Al Shehhi, Wafaa Eyaid. *Investigation*: Amjad Khan, Fahad A. Al Dihan, Bushra Al Shamsi, Maryam Al Shehhi, Wafaa Eyaid. *Data curation*: Amjad Khan, Bushra Al Shamsi, and Maryam Al Shehhi. *Validation and methodology*: Amjad Khan, Bushra Al Shamsi, Maryam Al Shehhi, Wafaa Eyaid. *Formal analysis*: Amjad Khan, Fahad A. Al Dihan, Bushra Al Shamsi, Maryam Al Shehhi, Wafaa Eyaid. *Supervision*: Bushra Al Shamsi, Maryam Al Shehhi, Wafaa Eyaid. *Writing—original draft*: Amjad Khan. *Writing—reviewing and editing*: all authors.

## FUNDING INFORMATION

The funders had no role in study design, data collection and analysis, decision to publish, or preparation of the manuscript. This study was financially supported by a fund from the ministry of National Guard Health Affairs, (MNGHA) Riyadh, Saudi Arabia, National genetic Center, The Royal Hospital, Ministry of Health (MOH), Sultanate of Oman, and King Abdullah University of Science and Technology (KAUST) through the baseline fund and the Award No. FCC/1/1976–25 and REI/1/4446–01 from the Office of Sponsored Research (OSR).

## CONFLICT OF INTEREST STATEMENT

The authors declare no conflict of interest.

## Supporting information


**TABLE S1.** Details of HGMD reported variants and reported phenotypes in POLR3A

## Data Availability

The data that support the findings will be available in [repository name] at [DOI/URL] following an embargo from the date of publication to allow for commercialization of research findings.
