# Supplementary material for: Further delineation of Wiedemann‐Rautenstrauch syndrome linked with POLR3A
Source: Mol Genet Genomic Med. 2024 Feb 13;12(3):e2274. doi: 10.1002/mgg3.2274 (PMC10958179; doi:10.1002/mgg3.2274)
Supplement: Supplementary file 1 — TABLE S1. Details of HGMD reported variants and reported phenotypes in POLR3A [file MGG3-12-e2274-s001.pdf]

Supplemental Table 1. Details of HGMD reported variants and reported phenotypes in *POLR3A*.

S.NO	DNA change	Amino acid change	Reported phenotype	References
1	c.3G>T	p.Met1Ile	Wiedemann-Rautenstrauch syndrome	Paolacci (2018) J Med Genet **55,** 837<br>Lessel (2018) *Hum Genet* **137:** 921
2	c.91C>T	p.Gln31Term	Spastic ataxia	Minnerop (2017) Brain **14,** 1561
3	c.169G>A	p.Asp57Asn	Hypomyelination, hypodontia & hypogonadotropic hypogonadism	Wolf (2014) Neurology **83,** 1898
4	c.200G>A	p.Arg67His	Leukodystrophy, POLR3-related	Ji (2018) PLoS One **13,** e0188869
5	c.272C>T	p.Pro91Leu	Hypomyelinating leukodystrophy	Daoud (2013) J Med Genet **50,** 194<br>Wolf (2014) *Neurology* **83:** 1898
6	c.325T>A	p.Cys109Ser	Spastic ataxia	Minnerop (2017) Brain **14,** 1561
7	c.328A>G	p.Lys110Glu	Leukodystrophy, POLR3-related	Musumeci (2022) Biomedicines **10,** 2276
8	c.346A>G	p.Met116Val	Hypomyelination, hypodontia & hypogonadotropic hypogonadism	Khalifa (2015) Eur J Med Genet **58,** 381
9	c.418C>T	p.Arg140Term	Leukodystrophy with oligodontia	Bernard (2011) Am J Hum Genet **89,** 415<br>Capalbo (2019) *PLoS Genet* **15:** e1008409
10	c.496G>A	p.Val166Ile	Hypomyelinating leukodystrophy	Daoud (2013) J Med Genet **50,** 194<br>Wolf (2014) *Neurology* **83:** 1898
11	c.592G>T	p.Glu198Term	Spastic ataxia	Infante (2020) J Neurol **267,** 324
12	c.760C>T	p.Arg254Term	Wiedemann-Rautenstrauch syndrome	Wambach (2018) Am J Hum Genet **103,** 968<br>Lessel (2018) *Hum Genet* **137:** 921<br>Hou (2020) *Proc Natl Acad Sci U S A* **117:** 3053
13	c.791C>T	p.Pro264Leu	Leukodystrophy, POLR3-related with striatal involvement without diffuse hypermyelination	Hiraide (2020) Brain Dev **42,** 363<br>Hiraide (2021) *Clin Genet* **100:** 40
14	c.919A>G	p.Met307Val	Varicella zoster virus infection	Ogunjimi (2017) J Clin Invest **127,** 3543
15	c.930G>C	p.Trp310Cys	Hypomyelination	Shimojima (2014) Brain Dev **36,** 315
16	c.1031G>T	p.Arg344Leu	Spastic paraplegia	D'Amore (2018) Front Neurol **9,**<br>Di Donato (2021) *Neurol Sci* **43:** 1071
17	c.1051C>T	p.Arg351Term	Leukodystrophy, POLR3-related	Perrier (2020) Neurol Genet **6,** e425<br>Sytsma (2021) *Neuropathology* **42:** 58
18	c.1067T>C	p.Leu356Pro	Spastic ataxia	Minnerop (2017) Brain **14,** 1561
19	c.1114G>A	p.Asp372Asn	Hypomyelination, hypodontia & hypogonadotropic hypogonadism	Bernard (2011) Am J Hum Genet **89,** 415<br>Wolf (2014) *Neurology* **83:** 1898
20	c.1160C>G	p.Ala387Gly	Hypomyelinating leukodystrophy	Daoud (2013) J Med Genet **50,** 194<br>Wolf (2014) *Neurology* **83:** 1898<br>Dinwiddie (2012) *J Gen Exome* **1:** 7
21	c.1186G>T	p.Val396Leu	Hypomyelination, hypodontia & hypogonadotropic hypogonadism	Wolf (2014) Neurology **83,** 1898
22	c.1310G>A	p.Arg437Gln	Varicella zoster virus infection	Ogunjimi (2017) J Clin Invest **127,** 3543
23	c.1360C>T	p.Leu454Phe	Spastic ataxia	Minnerop (2017) Brain **14,** 1561
24	c.1369G>A	p.Gly457Arg	Hypomyelinating leukodystrophy	Al Yazidi (2019) Mov Disord Clin Pract **6,** 155
25	c.1433C>G	p.Ala478Gly	Hypomyelination, hypodontia & hypogonadotropic hypogonadism	Wolf (2014) Neurology **83,** 1898
26	c.1451G>A	p.Arg484Gln	Leukodystrophy	Hiraide (2020) J Hum Genet **65,** 921
27	c.1531C>T	p.Gln511Term	Spastic ataxia	Minnerop (2017) Brain **14,** 1561
28	c.1544C>T	p.Ala515Val	Spastic ataxia	Minnerop (2017) Brain **14,** 1561
29	c.1674C>G	p.Phe558Leu	Tremor-ataxia with central hypomyelinating leukodystrophy	Bernard (2011) Am J Hum Genet **89,** 415<br>Wolf (2014) *Neurology* **83:** 1898
30	c.1672T>G	p.Phe558Val	Developmental delay, seizures, ataxia, hypertonia, spasticity	Monies (2019) Am J Hum Genet **104,** 1182
31	c.1676T>G	p.Phe559Cys	Growth hormone deficiency	Yu (2021) Front Endocrinol (Lausanne) **12,** 711991
32	c.1682G>A	p.Arg561Gln	Ataxia and spastic paraparesis	Rydning (2019) Brain **142,** e12
33	c.1681C>G	p.Arg561Gly	Hypomyelinating leukodystrophy	Neocleous (2020) Front Endocrinol (Lausanne) **11,** 626
34	c.1681C>T	p.Arg561Term	Leukodystrophy, POLR3-related	Perrier (2020) Neurol Genet **6,** e425
35	c.1721G>T	p.Gly574Val	Growth hormone deficiency	Yu (2021) Front Endocrinol (Lausanne) **12,** 711991
36	c.1724A>T	p.Lys575Met	Hypomyelination, hypodontia & hypogonadotropic hypogonadism	Khalifa (2015) Eur J Med Genet **58,** 381
37	c.1745G>A	p.Arg582His	Hypomyelination, hypodontia & hypogonadotropic hypogonadism	Khalifa (2015) Eur J Med Genet **58,** 381
38	c.1787C>T	p.Thr596Met	Spastic ataxia	Infante (2020) J Neurol **267,** 324
39	c.1797G>C	p.Gln599His	Hypomyelination, hypodontia & hypogonadotropic hypogonadism	Wolf (2014) Neurology **83,** 1898
40	c.1795C>A	p.Gln599Lys	Hypomyelination, hypodontia & hypogonadotropic hypogonadism	Wolf (2014) Neurology **83,** 1898<br>Musumeci (2022) *Biomedicines* **10:**
41	c.1800C>T	p.Ile600Ile	Wiedemann-Rautenstrauch syndrome	Paolacci (2018) J Med Genet **55,** 837
42	c.1804A>C	p.Ser602Arg	Hypomyelinating leukodystrophy	Daoud (2013) J Med Genet **50,** 194
**43**	**c.1895G>T**	**p.Cys632Phe**	**Microcephaly, developmental delay**	**This study**
44	c.1907C>A	p.Ser636Tyr	Hypomyelination, hypodontia & hypogonadotropic hypogonadism	Bernard (2011) Am J Hum Genet **89,** 415
45	c.1930G>A	p.Glu644Lys	Hypomyelination, hypodontia & hypogonadotropic hypogonadism	Wolf (2014) Neurology **83,** 1898

46	c.1935G>C	p.Leu645Phe	Hypomyelination, hypodontia & hypogonadotropic hypogonadism	Wolf (2014) Neurology **83,** 1898
47	c.2005C>G	p.Arg669Gly	Hypomyelination, hypodontia & hypogonadotropic hypogonadism	Wolf (2014) Neurology **83,** 1898
48	c.2005C>T	p.Arg669Term	Wiedemann-Rautenstrauch syndrome	Wambach (2018) Am J Hum Genet **103,** 968<br>Majethia (2021) *Am J Med Genet A* **185:** 1602<br>Smedley (2021) *N Engl J Med* **385:** 1868
49	c.2011T>C	p.Trp671Arg	Hypomyelinating leukodystrophy	Daoud (2013) J Med Genet **50,** 194<br>Wolf (2014) *Neurology* **83:** 1898
50	c.2015G>A	p.Gly672Glu	Tremor-ataxia with central hypomyelinating leukodystrophy	Bernard (2011) Am J Hum Genet **89,** 415<br>Wolf (2014) *Neurology* **83:** 1898<br>Pelletier (2021) *J Clin Endocrinol Metab* **106:** e660-e674<br>Capalbo (2019) *PLoS Genet* **15:** e1008409
51	c.2039T>C	p.Met680Thr	Hypomyelination, hypodontia & hypogonadotropic hypogonadism	Wolf (2014) Neurology **83,** 1898
52	c.2045G>A	p.Arg682Gln	Hypomyelination, hypodontia & hypogonadotropic hypogonadism	Wolf (2014) Neurology **83,** 1898<br>Harting (2020) *Neurogenetics* **21:** 121
53	c.2081G>A	p.Arg694His	Inborn error of metabolism	Barbosa-Gouveia (2021) Genes (Basel) **12,** 1262
54	c.2098A>T	p.Ile700Phe	Hypomyelination, hypodontia & hypogonadotropic hypogonadism	Wolf (2014) Neurology **83,** 1898
55	c.2120A>G	p.Gln707Arg	Varicella zoster virus infection	Ogunjimi (2017) J Clin Invest **127,** 3543
56	c.2119C>T	p.Gln707Term	Leukodystrophy, POLR3-related	Perrier (2020) Neurol Genet **6,** e425
57	c.2171G>A	p.Cys724Tyr	Leukodystrophy with oligodontia	Bernard (2011) Am J Hum Genet **89,** 415
58	c.2302C>T	p.Leu768Phe	Leukodystrophy, POLR3-related	La Piana (2016) Neurology **86,** 1622
59	c.2324A>T	p.Asn775Ile	Hypomyelination, hypodontia & hypogonadotropic hypogonadism	Bernard (2011) Am J Hum Genet **89,** 415<br>Wolf (2014) *Neurology* **83:** 1898
60	c.2325C>G	p.Asn775Lys	Leukodystrophy, type 7	Campopiano (2020) BMC Neurol **20,**
61	c.2350G>A	p.Gly784Ser	Hypomyelinating leukodystrophy	Tamura (2013) Rinsho Shinkeigaku **53,** 624<br>Vanderver (2020) *Ann Neurol* **88:** 264<br>Wolf (2014) *Neurology* **83:** 1898
62	c.2381A>C	p.Gln794Pro	Leukodystrophy, POLR3-related	La Piana (2016) Neurology **86,** 1622
63	c.2394T>A	p.Cys798Term	Spastic ataxia	Di Donato (2022) Neurol Sci **43,** 1071
64	c.2411T>C	p.Ile804Thr	Hypomyelination	Shimojima (2014) Brain Dev **36,** 315
65	c.2423G>A	p.Arg808Gln	4H syndrome	Tewari (2018) BMC Pediatr **18,**<br>Kaur (2021) *Clin Genet* **100:** 542
66	c.2422C>T	p.Arg808Term	Hypomyelinating leukodystrophy	Chen (2022) BMC Neurol **22,** 180
67	c.2425G>C	p.Val809Leu	Leukodystrophy, POLR3-related	Vanderver (2020) Ann Neurol **88,** 264
68	c.2438T>A	p.Phe813Tyr	Leukoencephalopathy	Lynch (2017) Brain **140,** 1204
69	c.2456C>T	p.Pro819Leu	Developmental delay, seizure, Wiedemann-Rautenstrauch syndrome,	This study
70	c.2471A>C	p.His824Pro	Hypomyelination, hypodontia & hypogonadotropic hypogonadism	Cohen (2020) Ann Hum Genet **84,** 11<br>Salinas (2020) *Am J Med Genet C Semin Med Genet* **184:** 876
71	c.2474C>G	p.Ser825Term	Wiedemann-Rautenstrauch syndrome	Paolacci (2018) J Med Genet **55,** 837
72	c.2521G>A	p.Gly841Ser	Cerebellar ataxia	Fogel (2014) JAMA Neurol **71,** 1237
73	c.2542T>C	p.Phe848Leu	Hypomyelination, hypodontia & hypogonadotropic hypogonadism	Wolf (2014) Neurology **83,** 1898
74	c.2547C>G	p.Phe849Leu	Hypomyelination, hypodontia & hypogonadotropic hypogonadism	Wolf (2014) Neurology **83,** 1898
75	c.2549A>G	p.His850Arg	Leukodystrophy, POLR3-related	La Piana (2016) Neurology **86,** 1622
76	c.2554A>G	p.Met852Val	Hypomyelination, hypodontia & hypogonadotropic hypogonadism	Bernard (2011) Am J Hum Genet **89,** 415<br>Di Donato (2021) *Neurol Sci* **43:** 1071<br>Campopiano (2020) *BMC Neurol* **20:** 258<br>Kyle (2021) *Parkinsonism Relat Disord* **85:** 23
77	c.2563C>T	p.Arg855Trp	Spastic ataxia	Benkirane (2021) Genet Med **23,** 2160
78	c.2618G>A	p.Arg873Gln	Hypomyelination, hypodontia & hypogonadotropic hypogonadism	Wolf (2014) Neurology **83,** 1898
79	c.2617C>T	p.Arg873Term	Neonatal progeroid syndrome	Jay (2016) Am J Med Genet A **170,** 3343<br>Minnerop (2017) *Brain* **140:** 1561<br>Capalbo (2019) *PLoS Genet* **15:** e1008409
80	c.2660A>T	p.Asp887Val	Hypomyelination, hypodontia & hypogonadotropic hypogonadism	Wolf (2014) Neurology **83,** 1898
81	c.2668G>T	p.Val890Phe	Leukoencephalopathy	Kunii (2018) Clin Genet **94,** 232<br>Furukawa (2021) *Front Neurol* **12:** 622355
82	c.2672G>A	p.Arg891Gln	Growth hormone deficiency	Yu (2021) Front Endocrinol (Lausanne) **12,** 711991
83	c.2671C>T	p.Arg891Term	Leukodystrophy, hypomyelinating	Fu (2017) Ultrasound Obstet Gynecol **51,** 493<br>Hiraide (2020) *Brain Dev* **42:** 363<br>Hiraide (2020) *Brain Dev* **42:** 363
84	c.2686G>A	p.Asp896Asn	Hypogonadotropic hypogonadism, idiopathic	Zhang (2021) Asian J Androl **23,** 288
85	c.2690T>A	p.Ile897Asn	Hypomyelination, cerebellar atrophy & corpus callosum hypoplasia	Saitsu (2011) Am J Hum Genet **89,** 644
86	c.2707G>A	p.Gly903Arg	Wiedemann-Rautenstrauch syndrome	Paolacci (2018) J Med Genet **55,** 837

87	c.2710G>A	p.Gly904Arg	Leukodystrophy, POLR3-related	La Piana (2016) Neurology **86,** 1622
88	c.2710G>T	p.Gly904Term	Spastic ataxia	Minnerop (2017) Brain **14,** 1561
89	c.2713G>A	p.Asp905Asn	4H leukodystrophy, striatal variant	Harting (2020) Neurogenetics **21,** 121
90	c.2722G>T	p.Asp908Tyr	Leukodystrophy, POLR3-related	Ji (2018) PLoS One **13,** e0188869
91	c.2774T>C	p.Leu925Pro	Leukodystrophy, POLR3-related	La Piana (2016) Neurology **86,** 1622
92	c.2809G>A	p.Glu937Lys	4H leukodystrophy, striatal variant	Harting (2020) Neurogenetics **21,** 121
93	c.2810A>T	p.Glu937Val	Hypomyelination, hypodontia & hypogonadotropic hypogonadism	Wolf (2014) Neurology **83,** 1898
94	c.2821A>C	p.Ser941Arg	Hypomyelination, hypodontia & hypogonadotropic hypogonadism	Wolf (2014) Neurology **83,** 1898
95	c.2830G>T	p.Glu944Term	Hypomyelination, hypodontia & hypogonadotropic hypogonadism	Bernard (2011) Am J Hum Genet **89,** 415<br>Wolf (2014) *Neurology* **83:** 1898
96	c.2993G>A	p.Arg998His	Developmental delay	Ganapathy (2019) J Neurol **266,** 1919
97	c.3013C>T	p.Arg1005Cys	Hypomyelination, hypodontia & hypogonadotropic hypogonadism	Bernard (2011) Am J Hum Genet **89,** 415<br>Saitsu (2011) *Am J Hum Genet* **89:** 644<br>Wolf (2014) *Neurology* **83:** 1898
98	c.3014G>A	p.Arg1005His	Hypomyelination, hypodontia & hypogonadotropic hypogonadism with late-onset growth hormone deficiency	Potic (2012) Arch Neurol **69,** 920<br>Capalbo (2019) *PLoS Genet* **15:** e1008409<br>Wolf (2014) *Neurology* **83:** 1898
99	c.3098T>C	p.Val1033Ala	Spastic ataxia	Minnerop (2017) Brain **14,** 1561
100	c.3115C>T	p.Gln1039Term	Spastic paraplegia	Travaglini (2018) Neurogenetics **19,** 111
101	c.3206G>A	p.Arg1069Gln	Wiedemann-Rautenstrauch syndrome	Paolacci (2018) J Med Genet **55,** 837
102	c.3205C>T	p.Arg1069Trp	Leukodystrophy, POLR3-related	La Piana (2016) Neurology **86,** 1622
103	c.3226G>A	p.Ala1076Thr	Leukodystrophy, POLR3-related	Arslan (2020) Brain Dev **42,** 6
104	c.3387C>A	p.Leu1129Leu	4H leukodystrophy, striatal variant	Harting (2020) Neurogenetics **21,** 121<br>Schlüter (2022) *Neurology* **98:** e912-e923<br>Schlüter (2022) *Neurology* **98:** e912-e923
105	c.3388G>A	p.Val1130Ile	Arthrogryposis, phenotypic modifier of	Bayram (2016) J Clin Invest **126,** 762
106	c.3392A>G	p.Lys1131Arg	Wiedemann-Rautenstrauch syndrome	Paolacci (2018) J Med Genet **55,** 837
107	c.3407G>A	p.Arg1136Gln	Hypomyelination, hypodontia & hypogonadotropic hypogonadism	Wolf (2014) Neurology **83,** 1898
108	c.3568C>T	p.Gln1190Term	Wiedemann-Rautenstrauch syndrome	Temel (2020) Eur J Hum Genet **28,** 1675
109	c.3655G>T	p.Gly1219Term	Ataxia and spastic paraparesis	Rydning (2019) Brain **142,** e12<br>Stranneheim (2021) *Genome Med* **13:** 40
110	c.3718G>A	p.Gly1240Ser	Hypomyelination, hypodontia & hypogonadotropic hypogonadism	Wolf (2014) Neurology **83,** 1898<br>Wan (2021) *Parkinsonism Relat Disord* **89:** 120<br>Lynch (2017) *Brain* **140:** 1204<br>Musumeci(2022) *Biomedicines* **10:**
111	c.3721G>A	p.Val1241Met	Neurodevelopmental regression, generalized dystonia and metabolic acidosis	Zanette (2020) Neurol Genet **6,** e521
112	c.3733C>T	p.Arg1245Term	Spastic ataxia	Di Donato (2022) Neurol Sci **43,** 1071
113	c.3745A>C	p.Asn1249His	Hypomyelination, hypodontia & hypogonadotropic hypogonadism	Terao (2012) J Neurol Sci **320,** 102
114	c.3781G>A	p.Glu1261Lys	Hypomyelinating leukodystrophy	Daoud (2013) J Med Genet **50,** 194<br>Minnerop(2017) *Brain* **140:** 1561
115	c.3820A>C	p.Thr1274Pro	Hypomyelinating leukodystrophy	Di Bella (2021) Eur J Neurol **28,** 934
116	c.3858C>A	p.His1286Gln	Hypomyelination, hypodontia & hypogonadotropic hypogonadism	Stranneheim (2021) Genome Med **13,** 40
117	c.3874G>A	p.Asp1292Asn	Wiedemann-Rautenstrauch syndrome	Paolacci (2018) J Med Genet **55,** 837
118	c.3882G>C	p.Met1294Ile	Transposition of the great arteries	Jin (2017) Nat Genet **49,** 1593<br>Edwards (2020) *JACC Basic Transl Sci* **5:** 376
119	c.3986T>C	p.Phe1329Ser	Leukodystrophy, hypomyelinating, 7, with or without oligodontia and/or hypogonadotropic hypogonadism	French (2022) HGG Adv **3,** 100113
120	c.3991G>A	p.Ala1331Thr	Hypomyelination, hypodontia & hypogonadotropic hypogonadism with late-onset growth hormone deficiency	Potic (2012) Arch Neurol **69,** 920<br>Wolf (2014) *Neurology* **83:** 1898
121	c.4003G>A	p.Gly1335Arg	Wiedemann-Rautenstrauch syndrome	Paolacci (2018) J Med Genet **55,** 837
122	c.4006C>T	p.Gln1336Term	Hypomyelination, hypodontia & hypogonadotropic hypogonadism	Bernard (2011) Am J Hum Genet **89,** 415
123	c.4044C>G	p.Ile1348Met	Hypomyelinating leukodystrophy	Chen (2022) BMC Neurol **22,** 180
124	c.4073G>A	p.Gly1358Glu	Spastic ataxia	Di Donato (2022) Neurol Sci **43,** 1071<br>Galatolo (2021) *Int J Mol Sci* **22:**
125	c.4108C>T	p.Pro1370Ser	Developmental disorder	Turner (2019) Am J Hum Genet **105,** 1274
126	c.490+1G>A	-	Wiedemann-Rautenstrauch syndrome	Wambach (2018) Am J Hum Genet **103,** 968
127	c.645+312C>T	-	Leukodystrophy	Hiraide (2020) J Hum Genet **65,** 921
128	c.1048+1G>A	-	Leukodystrophy, POLR3-related	La Piana (2016) Neurology **86,** 1622
129	c.1048+5G>T	-	Spastic ataxia	Minnerop (2017) Brain **14,** 1561<br>Paolacci (2018) *J Med Genet* **55:** 837<br>Harting (2020) *Neurogenetics* **21:** 121

130	c.1290-2A>G	-	Spastic ataxia	Minnerop (2017) Brain **14,** 1561
131	c.1289+3A>C	-	Leukodystrophy, POLR3-related	La Piana (2016) Neurology **86,** 1622
132	c.1572+1G>A	-	Wiedemann-Rautenstrauch syndrome	Wambach (2018) Am J Hum Genet **103,** 968
133	c.1771-7C>G	-	Spastic ataxia	Minnerop (2017) Brain **14,** 1561<br>Majethia (2021) *Am J Med Genet A* **185:** 1602<br>Wan (2021) *Parkinsonism Relat Disord* **89:** 120<br>Perrier (2020) *Neurol Genet* **6:** e425
134	c.1771-6C>G	-	Leukodystrophy, POLR3-related	La Piana (2016) Neurology **86,** 1622<br>Harting (2020) *Neurogenetics* **21:** 121<br>Zanette (2020) *Neurol Genet* **6:** e521<br>Hiraide (2020) *Brain Dev* **42:** 363
135	c.1770+5G>C	-	Hypomyelinating leukodystrophy	Yan (2021) J Hum Genet **66,** 761
136	c.1909+18G>A	-	Leukodystrophy with oligodontia	Bernard (2011) Am J Hum Genet **89,** 415<br>Paolacci (2018) *J Med Genet* **55:** 837<br>Jay (2016) *Am J Med Genet A* **170:** 3343<br>Yang (2019) *Chin Med J (Engl)* **132:** 1879
137	c.1909+22G>A	-	Leukodystrophy, POLR3-related	La Piana (2016) Neurology **86,** 1622<br>Paolacci (2018) *J Med Genet* **55:** 837<br>Galatolo (2021) *Int J Mol Sci* **22:**<br>Riso (2021) *Brain Sci* **11:**
138	c.2248-1G>C	-	Inborn error of metabolism	Barbosa-Gouveia (2021) Genes (Basel) **12,** 1262
139	c.2247+2T>G	-	Spastic ataxia	Minnerop (2017) Brain **14,** 1561
140	c.2617-1G>A	-	Hypomyelination, hypodontia & hypogonadotropic hypogonadism	Bernard (2011) Am J Hum Genet **89,** 415<br>Paolacci (2018) *J Med Genet* **55:** 837<br>Minnerop (2017) *Brain* **140:** 1561<br>Wolf (2014) *Neurology* **83:** 1898
141	c.2788-2A>T	-	Spastic ataxia	Di Donato (2022) Neurol Sci **43,** 1071
142	c.2988+1G>T	-	Hypomyelination, hypodontia & hypogonadotropic hypogonadism	Wolf (2014) Neurology **83,** 1898
143	c.3243-2A>G	-	Wiedemann-Rautenstrauch syndrome	Wambach (2018) Am J Hum Genet **103,** 968
144	c.3337-11T>C	-	Wiedemann-Rautenstrauch syndrome	Paolacci (2018) J Med Genet **55,** 837<br>Sukenik-Halevy (2022) *Prenat Diagn* **42:** 717<br>Temel (2020) *Eur J Hum Genet* **28:** 1675
145	c.3337-5T>A	-	Wiedemann-Rautenstrauch syndrome	Wambach (2018) Am J Hum Genet **103,** 968<br>Lessel (2018) *Hum Genet* **137:** 921
146	c.3337-1G>A	-	Wiedemann-Rautenstrauch-like progeroid Syndrome	Lessel (2018) Hum Genet **137,** 921
147	c.3336G>A	-	Intrauterine growth retardation, lipodystrophy, muscular hypotonia and facial anomalies	Lessel (2022) Am J Med Genet A **188,** 216<br>Lessel (2021) *Am J Med Genet A* **188:** 216
148	c.3593A>G	-	Spastic ataxia	Morales-Rosado (2020) Mol Genet Genomic Med **8,** e1341
149	c.4025-1G>A	-	4H leukodystrophy, striatal variant	Harting (2020) Neurogenetics **21,** 121<br>van der Ven (2021) *Clin Genet* **100:** 766
150	c.*18C>T	-	Wiedemann-Rautenstrauch syndrome	Paolacci (2018) J Med Genet **55,** 837
151	c.367_369delAAG	p.Lys123del	Hypomyelination, hypodontia & hypogonadotropic hypogonadism	Wolf (2014) Neurology **83,** 1898<br>Kyle (2021) *Parkinsonism Relat Disord* **85:** 23
152	c.601delA	p.Ile201Leufs*18	Leukodystrophy, POLR3-related	Perrier (2020) Neurol Genet **6,** e425
153	c.1378_1380delGTG	p.Val460del	Ataxia and spastic paraparesis	Rydning (2019) Brain **142,** e12

154	c.1650_1661del12	p.Leu551_Leu554del	Leukodystrophy, POLR3-related	Yoon Han (2022) Clin Chim Acta **533,** 15
155	c.2376_2377delAT	p.Ser793Thrfs*21	Inborn error of metabolism	Barbosa-Gouveia (2021) Genes (Basel) **12,** 1262
156	c.2472delC	p.Ser825Glnfs*18	Spastic ataxia	Minnerop (2017) Brain **14,** 1561
157	c.2554delA	p.Met852Trpfs*7	Spastic ataxia	Minnerop (2017) Brain **14,** 1561<br>Smedley (2021) *N Engl J Med* **385:** 1868
158	c.2561delG	p.Gly854Alafs*5	Spastic ataxia	Minnerop (2017) Brain **14,** 1561
159	c.2611delA	p.Met871Cysfs*18	Leukodystrophy, POLR3-related	Wu (2019) BMC Pediatr **19,** 289
160	c.3201_3202delGC	p.Arg1069Aspfs*2	Spastic paraplegia	D'Amore (2018) Front Neurol **9,**<br>Riso (2021) *Brain Sci* **11:**<br>Di Donato (2021) *Neurol Sci* **43:** 1071
161	c.3583delG	p.Asp1195Ilefs*47	Leukodystrophy, POLR3-related	Perrier (2020) Neurol Genet **6,** e425<br>Molina-Ramírez (2021) *J Med Genet* **59:** 393
162	c.3772_3773delCT	p.Leu1258Glyfs*12	Wiedemann-Rautenstrauch syndrome	Paolacci (2018) J Med Genet **55,** 837<br>Báez-Becerra (2020) *Mech Ageing Dev* **192:** 111360
163	c.3944_3945delTG	p.Val1315Alafs*7	Spastic ataxia	Minnerop (2017) Brain **14,** 1561
164	c.441dupT	p.Asp148*	Hypomyelination, hypodontia & hypogonadotropic hypogonadism	Wolf (2014) Neurology **83,** 1898
165	c.659_661dupCCT	p.Pro220_Leu221insSer	Hypomyelinating leukodystrophy	Yan (2021) J Hum Genet **66,** 761
166	c.1301dupA	p.Tyr434*	Hypomyelinating leukodystrophy	Daoud (2013) J Med Genet **50,** 194<br>Wolf (2014) *Neurology* **83:** 1898
167	c.1740dupA	p.Val581Serfs*28	Hypomyelinating leukodystrophy	Daoud (2013) J Med Genet **50,** 194<br>Wolf (2014) *Neurology* **83:** 1898
168	c.1993dupT	p.Tyr665Leufs*11	Spastic ataxia	Infante (2020) J Neurol **267,** 324
169	c.3739_3741dupACC	p.Thr1247dup	Tremor-ataxia with central hypomyelinating leukodystrophy	Bernard (2011) Am J Hum Genet **89,** 415<br>Wolf (2014) *Neurology* **83:** 1898
170	c.3839dupT	p.Met1280Ilefs*20	Spinocerebellar ataxia	Galatolo (2021) Int J Mol Sci **22,**
171	c.552_553delAGinsT	p.Lys184Asnfs*35	Hypomyelination, hypodontia & hypogonadotropic hypogonadism	Wolf (2014) Neurology **83,** 1898
172	c.646-666_1185+865del2370	p.Glu216_Lys395del	Spastic ataxia	Infante (2020) J Neurol **267,** 324
